# Developing Kaspar: A Humanoid Robot for Children with Autism

**DOI:** 10.1007/s12369-019-00563-6

**Published:** 2019-07-08

**Authors:** Luke J. Wood, Abolfazl Zaraki, Ben Robins, Kerstin Dautenhahn

**Affiliations:** School of Computer Science, University of Hertfordshire, Hatfield, UK

**Keywords:** Autism therapy, Humanoid social robots, User-centred design, Autonomous systems, Cognitive architecture

## Abstract

In the late 1990s using robotic technology to assist children with Autistic Spectrum Condition (ASD) emerged as a potentially useful area of research. Since then the field of assistive robotics for children with ASD has grown considerably with many academics trialling different robots and approaches. One such robot is the humanoid robot Kaspar that was originally developed in 2005 and has continually been built upon since, taking advantage of technological developments along the way. A key principle in the development of Kaspar since its creation has been to ensure that all of the advances to the platform are driven by the requirements of the users. In this paper we discuss the development of Kaspar’s design and explain the rationale behind each change to the platform. Designing and building a humanoid robot to interact with and help children with ASD is a multidisciplinary challenge that requires knowledge of the mechanical engineering, electrical engineering, Human–Computer Interaction (HCI), Child–Robot Interaction (CRI) and knowledge of ASD. The Kaspar robot has benefited from the wealth of knowledge accrued over years of experience in robot-assisted therapy for children with ASD. By showing the journey of how the Kaspar robot has developed we aim to assist others in the field develop such technologies further.

## Introduction

**Fig. 1:**
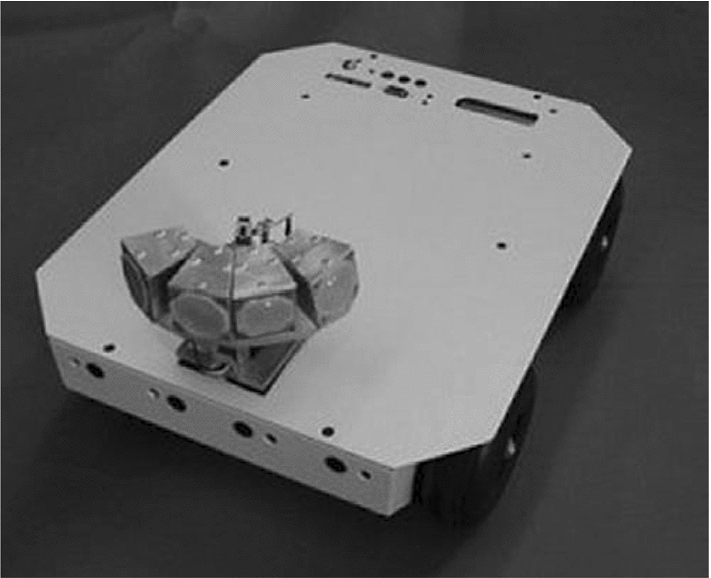
Mobile robot

**Fig. 2:**
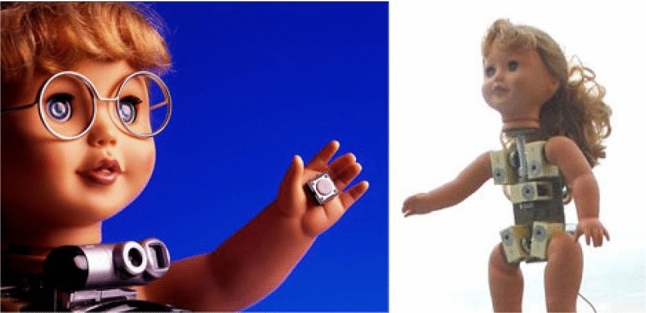
Robota

Investigating how robots could potentially be used as assistive tools for children with Autistic Spectrum Disorder (ASD) emerged as a field of research in the late 1990s with Dautenhahn and Werry [1, 2] conducting some of the first studies in this area. Research in assistive robotics for children with ASD was initially conducted with small mobile robots (Fig. 1), but was soon followed up by the possibility of humanoid robots. One of the first humanoid robots to be used in an assistive capacity for children with ASD was a small robotic doll called Robota (Fig. 2) [3]. The Robota robot possessed 5 Degrees of Freedom (DOF). Robota took the form of a pretty doll and could move its limbs (arms and legs) and head to interact with the children. This work was soon followed up in 2004 by Kozima et al. [4] who investigated how a child with ASD interacted with a more complex humanoid robot called Infanoid that possessed 29 DOF. In contrast to Robota, the face of Infanoid was not human-like in appearance. To establish the impact of appearance on children with ASD and how this affects interactions Robins et al. conducted a study in which a mime artist performed a set of pre-determined moves (like a robot). The mime artist was either in plain clothes or dressed up as a silver robot complete with facial makeup. Robins et al. found that appearance can have a substantial impact on the children’s desire to interact [5] and later followed this up with a study using the Robota robot which provided further evidence for this conclusion [6]. The lessons learnt from both of these studies were considered during the development of the humanoid robot Kaspar which is the subject of this article.

## The Kaspar Robot

**Fig. 3:**
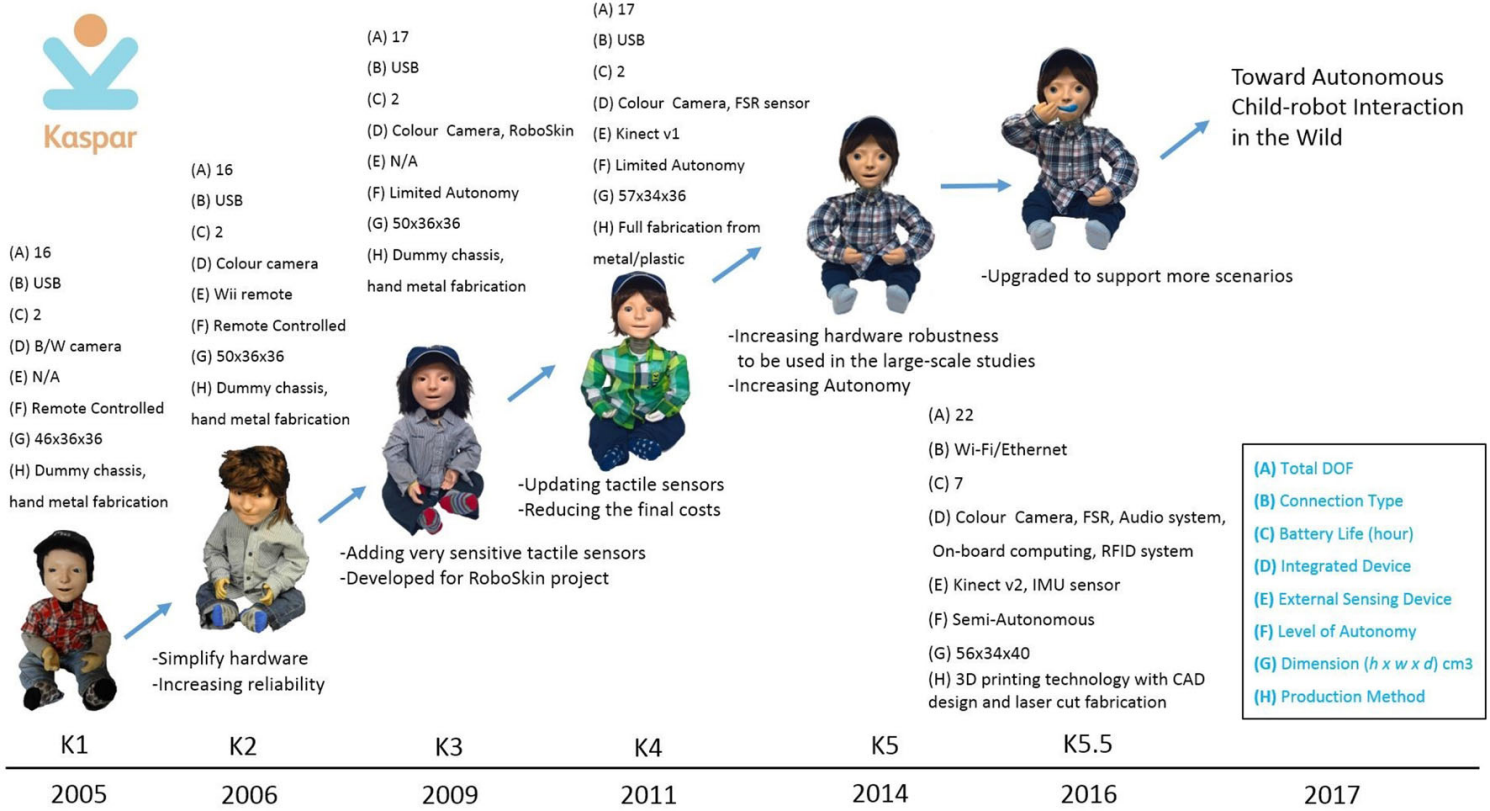
Kaspar versions from 2005 onwards

The Kaspar robot was originally developed in 2005 by Blow et al. [7] as a Human–Robot Interaction research platform. Because of the simplified human-like appearance of Kaspar, the robot was quickly adopted to investigate how it could be used as a therapeutic device for children with ASD [6]. Since the creation of the first Kaspar robot in 2005 there have subsequently been five more generations of the robot developed (Fig. 3). The development of the robot over this period has focused on improving the robot’s functionality and usability as a therapeutic and educational tool for researchers, teachers and therapists working with children with ASD. In addition to the robot’s primary application, Kaspar has also been used in other human–robot interaction studies [8, 9].

Since the initial creation of Kaspar there have been numerous technological advances and the accessibility of sophisticated manufacturing has increased due in part to the expiration of major 3D printing patents in recent years [10, 11]. The first Kaspar prototype (K1), constructed in 2005, was manufactured by hand fabricating metal parts for the robot. This robot was equipped with modest sensing capability and every aspect of the robot’s behaviour had to be controlled remotely by a human operator during child–robot interactions. In contrast, the most recent iteration of Kaspar (K5.5) has been designed in CAD software and produced using modern manufacturing methods such as laser cutting and 3D printing. Using this approach has made the robot more robust and has also allowed for more robots to be produced with greater ease. Further to this, the K5.5 robots are also equipped with hardware and software which is much more advanced, thus enabling reliable and reproducible semi-autonomous child–robot interactions. The K5.5 robots have been used with over 300 children in a number of different capacities, ranging from programming classes with Typically Developing (TD) children to therapeutic sessions with interaction games that focus on skills such as Visual Perspective Taking (VPT) with children with ASD [12]. The most recent developments on the Kaspar platform have allowed us to create games that use advances in sensing technology and computing techniques to perceive the environment and make decisions about the observed social cues and events which are employed to facilitate interactions by activating appropriate body gestures, facial expressions and vocal communication within the robot.

**Fig. 4:**
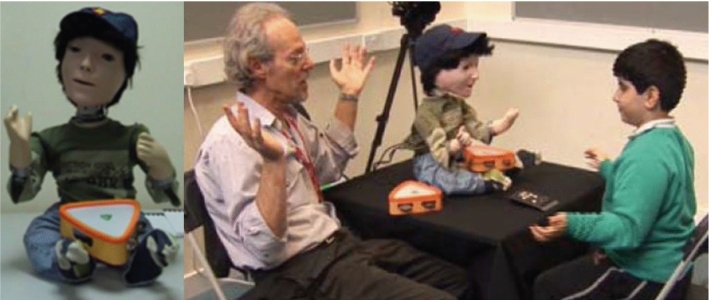
K1 robot during imitation game

The following sections provide a detailed explanation of the developmental journey of the Kaspar robot since 2005 and how this has been influenced by user requirements, technological advances and accessibility of modern manufacturing techniques.

### The First Kaspar Robot (K1)

The first Kaspar robot (K1, Fig. 4) designed in 2005 by M. Blow et al. was intended to be a Human–Robot Interaction platform for a short term research project [7]. Since Kaspar has a human-like face with realistic but simplified features (i.e. it has a nose, eyes, a mouth etc. but no facial hair or skin colouration), the robot was soon adopted to study how such robots could be used as therapeutic devices for children with ASD [6]. At that time, the Kaspar robot presented a leap in the field of assistive robotics for children with ASD as the Robota robot (Fig. 2), that had primarily been used in this field before, only had 5 DOF, which significantly limited the gestures, body movements and postures that the robot could adopt, did not possess and expressive face, and was quite small (doll-sized). By contrast the K1 Kaspar robot possessed 16 DOF and measured approximately 46cm in height by 36cm width and 36cm depth with an expressive face capable of producing simple but realistic expressions. The construction of the K1 robot was based around a shop window dummy body of a 2-year old child for the main chassis with the robotic elements being constructed from off the shelf RC servos and hand fabricated metal parts. The servos used for the robot were relatively inexpensive as one of the design principles for the robot was to produce it for less than 2000 euros. The head of the robot in particular used micro servos for the eyes because of the compact nature of the space required to fit the components [7]. The K1 robot was powered by a 12VDC low voltage lead acid gel battery with a run time of approximately 2 h from a 4 h charge time. This first version of the Kaspar robot was used for a number of activities and games that encouraged skills such as turn taking to the exploration of facial expressions [13–16]. Further to this the robot was also used to explore the possibility of conducting robot-mediated interviews with children [17–20] and to explore interaction dynamics and gestures in human–humanoid drumming experiments [21, 22] The second implementation of the Kaspar robot K1-L was built in 2006 and was similar in construction to the K1 version but was a much larger robot using a 6-year old child dummy as a chassis measuring approximately 123cm in height by 30 cm wide and 57 cm deep. This version of Kaspar was mounted on a desktop PC and was equipped with an extra DOF in each arm, providing the robot with a total of 18 DOF. Because this version of the Kaspar robot was primarily intended as an early software development platform for the Robotcub project [23] it was also furnished with additional sensing capabilities including a laser depth camera and joint feedback sensors. Rather than being used for children with ASD the purpose of this version of Kaspar was for cognitive and AI software developments, testing and HRI studies [24]. After this version of Kaspar all future iterations of the robot were designed to be used specifically with children with ASD in schools or home environments and as such were designed to be easily transported and set up.

**Fig. 5:**
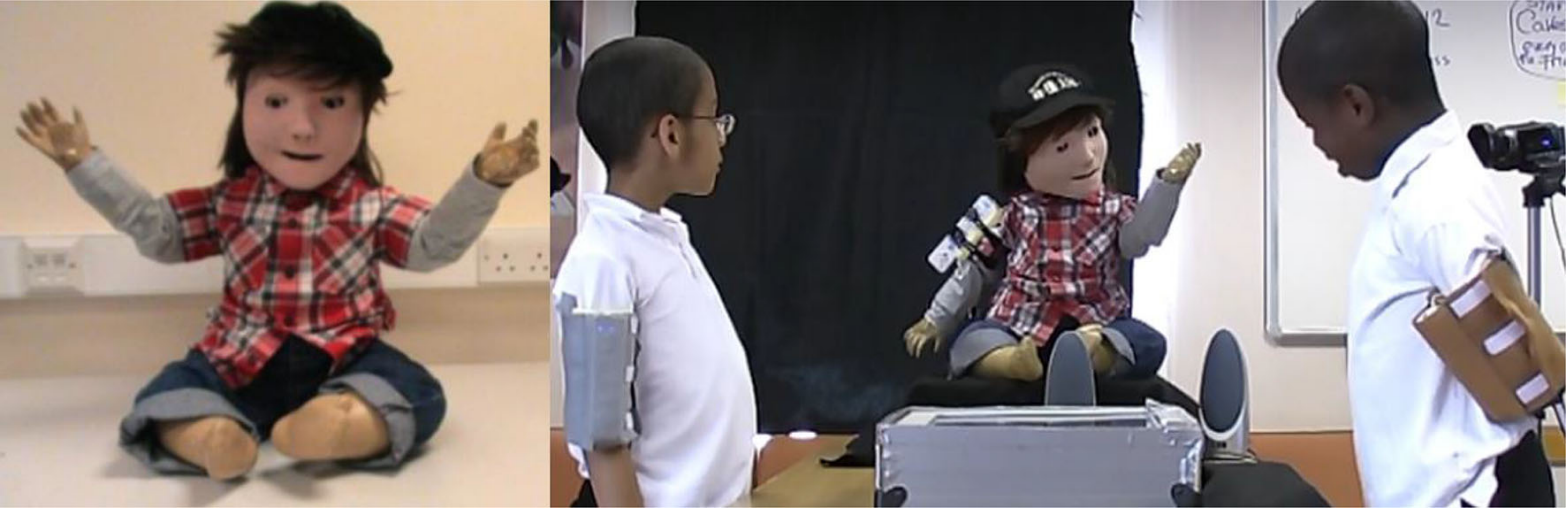
K2 robot during fully autonomous triadic game

### The Second Kaspar Robot (K2)

The K1 robot was very successful in its ability to attract and maintain the attention and interest of many children with ASD who had interacted with the robot. It provided more advanced interaction possibilities than the previously used Robota robot. Thus, a second implementation of Kaspar (K2, Fig. 5) was developed in 2006 by M. Walters to facilitate further research investigating the potential applications using such a robot with children with ASD [25]. The K2 robot possessed a specification similar to the K1 robot—with 16 DOF and a comparable overall size, measuring approximately 50 cm in height by 36 cm width and 36 cm depth. The main upgrades to K2 were simplifications to the hardware in the robot’s head to make the robot more robust and the inclusion of colour cameras in the eyes as an upgrade to the black and white cameras of K1. One of the most notable developments implemented on K2 was the integration of a Nintendo wii [26] remote to facilitate a fully autonomous collaborative game for children with ASD to play dyadic and triadic games [27, 28]. The game used a small monitor to provide visual feedback and wii remotes strapped to the arms of each player. In order to earn rewards in the game the participants had to collaborate and work together. Using this setup two studies were conducted, a dyadic study [27], evaluating how children play with a human player compared to playing with a humanoid robot, and a triadic long-term study [28] where the children played triadically with the Kaspar robot with all players (including Kaspar) having an equal role in the game. Pre- and post- assessments where pairs of children played the game with each other, without the robot, showed improvements in the children’s collaborative skills. However, there were some important lessons learned from this study that were taken forward to future developments of the Kaspar platform. Because Kaspar was operating as a fully autonomous system in this study, it greatly limited the type of children that could be worked with. Previously, the studies conducted with Kaspar had focused on working with children on the lower functioning end of the ASD spectrum. However, the constrained nature of a fully autonomous setup made it impossible to work with low functioning children which requires much more flexibility of adjusting the games on the fly. Also, this setup required the robot’s external sensors, i.e. the wii remotes, to be attached to the children’s arms which is not ideal because these devices can move and become loose. They also have the capacity to distract the children, and for some children with ASD it can be uncomfortable to be required to wear extra sensors/clothing/devices on their bodies. Taking these factors into consideration all future developments to the Kaspar platform focused on semi-automation rather than full automation because this would make the system more flexible as well as useful to a wider range of users. Further to this, relying on sensors that the children would have to wear was avoided in the future.

### The Third Kaspar Robot (K3)

**Fig. 6:**
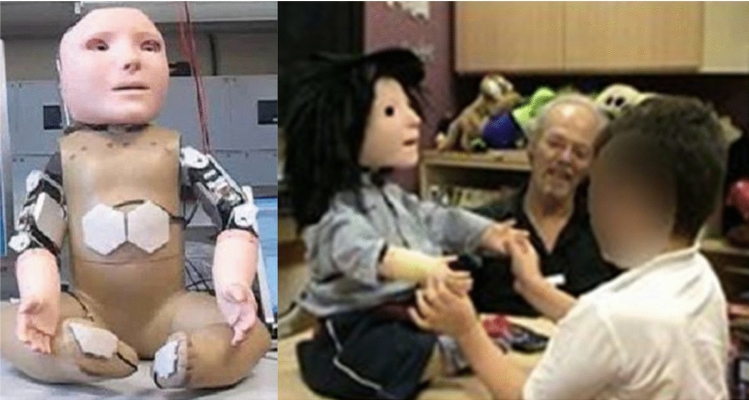
K3 robot during tactile interaction

Driven by a European project called ROBOSKIN, in 2009 the third version of the Kaspar robot was constructed by M. Walters (K3, Fig. 6). Identical in size to K2, K3 measured approximately 50 cm in height by 36 cm width and 36 cm depth, with an extra DOF giving the robot 17 DOF in total. The aptly named ROBOSKIN project was focused on developing robotic skin capable of facilitating tactile interaction with robots [29, 30]. The K3 was largely based on the K2 but included a number of new features based on what had been learnt from previous versions of the robot. Unsurprisingly the most notable feature added to the K3 was the addition of tactile skin patches strategically placed on the robots feet, hands and chest to enable tactile interaction and to assist children learn about what is and is not appropriate tactile interaction. The tactile sensors on the robot were called ROBOSKIN and used distributed pressure sensors based on capacitive technology. The transducer consists of a soft dielectric sandwiched by electrodes. When force is applied to the sensor patch the distance between the electrodes change, causing the capacitance to change accordingly. The ROBOSKIN system in particular was constructed with a number of tactile elements (taxels) geometrically organized in interconnected modules of triangular shape. The flexible PCB was covered by a layer of silicone foam and acted as a deformable dielectric. The additional DOF integrated into the robot was placed in the torso and enabled the robot to turn left and right creating the possibility of more complex gestures. Having this additional DOF allowed the robot to turn away, e.g. when a child had hit the robot. Since Kaspar is not a mobile robot, this was an important additional feature. It allowed us to teach children with ASD about socially appropriate tactile interaction [31]. Children with ASD may either crave or avoid tactile interaction (named hyper- and hyposensitivity), so this facilitated the development of a range of tactile interaction scenarios. In addition to the ROBOSKIN and the torso, a speaker was integrated into K3 which assisted in creating a stronger effect of the robot itself speaking as opposed to the sound coming from a slightly displaced location, e.g. a laptop or additional speaker. Studies making use of the ROBOSKIN installed on Kaspar showed that the sensors could be useful in facilitating robot assisted play and had the potential to expand the repertoire activities and functions that the robot could perform in an assistive capacity for children with ASD [32, 32–37]. Because of the success of the upgrades to the K3 robot in terms of their ability to facilitate more complex and useful scenarios in a more realistic way, all future versions of the Kaspar robot would include tactile sensors, an on-board speaker and the additional DOF to enable the torso movement.

### The Fourth Kaspar Robot (K4)

**Fig. 7:**
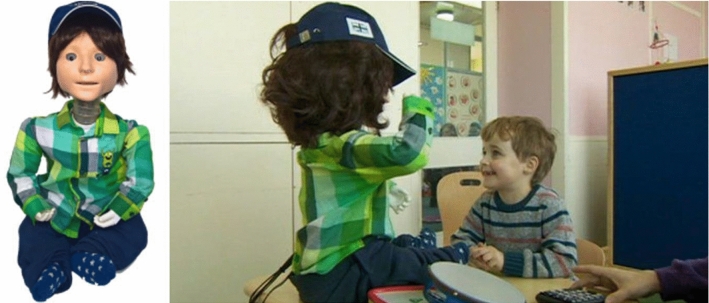
K4 robot interacting with child in a nursery school

Based on the positive results of the previous studies with the Kaspar robots since 2005, indicating that robots such as Kaspar could assist in helping children with ASD e.g. develop their turn taking skills, collaborative skills and tactile social interaction skills [38], the first small production run of 7 Kaspar robots was designed and developed by Merlin Systems Corporation Limited in 2011 (K4, Fig. 7), contracted by the University of Hertfordshire. The purpose of these robots was to enable more research to be conducted into how the robot could be used to help children with ASD, but also for the Kaspar robot to be used outside of the immediate research team with teachers in schools. The K4 robot measured approximately 57 cm in height by 34 cm width and 36 cm depth. Similar to the specification of the K3 the K4 had 17 DOF and included a speaker in the body of the robot. The K4 robot was inspired by the K2 and K3 robots but was totally redesigned. Rather than using a dummy body as a chassis like all previous versions, this version was engineered and fabricated from scratch using plastic and metal parts. Because the tactile sensors were used to such positive effect in the K3 robot [37], the K4 robots also included 10 tactile sensors. However, the sensors integrated into the robot were Force Sensing Resistor (FSR) sensors and were simpler, but much more reliable and cheaper than the initial prototype ROBOSKIN sensors available for K3. Using these FSR sensors allowed the component cost of the robot to be kept below 2000 euros whilst still providing adequate functionality for tactile interaction. To utilise these sensors the software that runs the Kaspar robot required further development. The initial integration of the FSR sensors was conducted by Barbadillo [39] but was later refined by O. Novanda to filter the electrical noise from the sensors. Another change on the K4 robot was the type of servo used. The K4 was developed using the smart Dynamixel AX-12 servos where previous generations used much more basic servos. Because these servos were far superior to the servos used on previous robots, the K1, K2 and K3 robots were all upgraded with these servos too, to increase robustness and accuracy of the robot’s joints. The K4 robots were also equipped with a small on-board PC complete with Wi-Fi and Ethernet connectivity. However, because the software that these robots came with was not sufficiently reliable or user friendly it was later replaced with a USB serial connection that had been used with previous versions. A number of studies were conducted with these robots and they allowed research to be conducted outside of the immediate research team in the university because of the number of robots produced [40, 41].

### The Fifth Kaspar Robot (K5, K5.5)

**Fig. 8:**
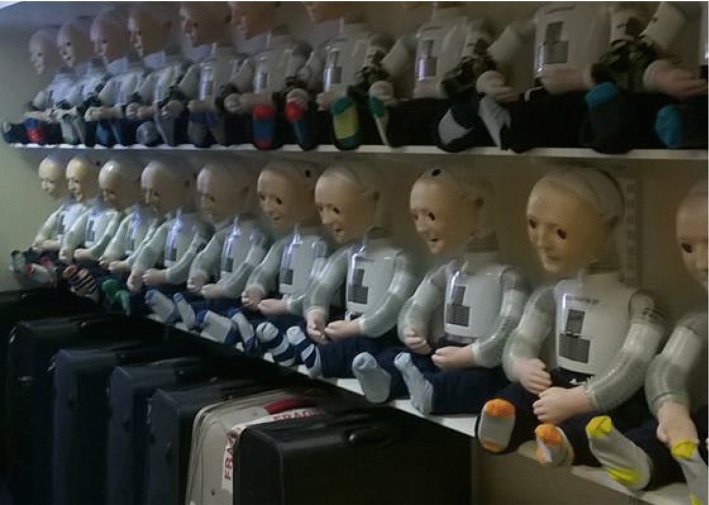
Production run of 20 K5 robots

The K5 robot (Fig. 8) was designed from scratch by L. J. Wood and M. Walters in 2014, with 20 robots being produced. The K5 was slightly bigger than previous versions to accommodate the new features and as such it measures approximately 56cm in height by 34 cm width and 40 cm depth. Lessons learned from all previous versions of the robot along with modern design and manufacturing methods were used to radically re-design the platform. Because a production run of 20 robots was planned, the design of the robot also needed to suit this level of manufacturing. Therefore CAD design, 3D printing, laser cutting and vacuum forming were used. This was the first version of Kaspar that was suitable to be used by parents and teachers or therapists independently without a researcher present.

- *Servos*—The K5 robot possesses 22 DOF with 3 DOF in each eye/eyelid, 2 DOF in the mouth, 3 DOF in the neck, 5 DOF in each arm and 1 DOF in the torso. The DOF in the eyes/eyelids of the robot used the Hitec HS-82MG servos whilst the more substantial joints in the robot used the Dongbu Robot Herkulex drs-0101 and drs-0201 servos. The Herkulex drs servos were chosen because of their small form and compliance feature. The servos could be programmed to provide an elastic response to external force, meaning that if a child moved the arm of the robot manually and forced it, the servo would not break as they would have on previous models. Using these servos made the robot much more reliable and suitable to tactile interaction.
- *Sensors*—The K5 uses 15 FSR sensors to facilitate tactile interaction which are placed as follows: 2 in each hand with one on the palm and another on the back of the hand, 1 on each of the arms, 1 on each of the legs, 1 on each of the feet, 1 on the chest and 4 in the face of the robot.
- *Connectivity*—This version of Kaspar was the first to utilise Wi-Fi connectivity and was therefore no longer required to be physically tethered to a computer—an important feature to remove the hazard of users tripping over wires.
- *Power*—The robot is powered by two 12 v 7Ah Lithium Iron Phosphate batteries which can last for up to 7 h. The recharge time of these batteries is 6 h, but has the capacity to be much faster with a 7 amp charger.
- *Speaker*—The speaker of the robot was mounted in the head to help create the illusion that the sound is coming from Kaspar’s mouth.
- *Concealment*—Because this version of Kaspar was to be used directly by parents and teachers it was essential to ensure all wires, servos and metal parts were concealed, as they were not in previous iterations of Kaspar. The design of the K5 concealed as many parts of the robot as possible to eliminate the potential for small fingers getting caught in gaps and to make the robot more robust. An example of this is the construction of the hands. The FSR sensors were placed on the 3D printed core then covered by a silicon skin which protected the sensors and provided the hands with a pleasant feel. The arm and neck joints of the K5 were shielded with bellows (flexible covers) that were designed in CAD and 3D printed using NinjaFlex a thermoplastic polyurethane (TPU) material. Note, in order to maintain the introduction of Kaspar as a robot, not a ‘small child’, we covered e.g. the robot’s neck with transparent flexible, 3D printed covers, so that the robotic nature of the robot was clearly visible to the children and adults present.

**Fig. 9:**
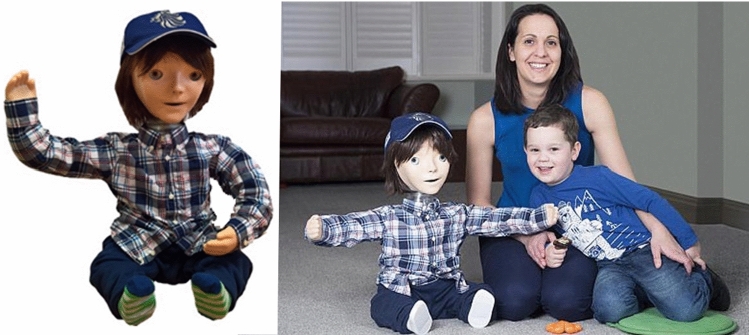
K5.5 robot playing VPT game with child

Since 2014 a number of hardware and software upgrades have been made to the K5 platform to improve the functionality and usability of the robot constituting the K5.5 robot (Fig. 9). Part of the upgrade to Kaspar was the integration of an RFID reader which is ideal for the Kaspar platform as it is an inexpensive and reliable technology. Currently the RFID reader is used to switch between game modalities on the robot without having to use the PC, but there are plans to use this technology in the future to enable the robot to detect tagged toys and build games around these toys. This technology is well suited to creating a level of autonomy within the robot due to its robustness. In addition to the RFID technology the hands of K5.5 included upgraded hands with strong neodymium magnets to enable the robot to hold objects placed in its hands. This upgrade has already had an impact in a nursery school where the Kaspar robot has been used with magnetic accessories including a fork, spoon, hairbrush and toothbrush. The staff from the nursery have reported that this feature has been useful for encouraging some children with ASD to eat by simulating eating with the magnetic spoon and fork, and it can be used to teach about personal hygiene, e.g. brushing the teeth or combing the hair. Those upgrades on the K5.5 robot have all been driven by the user requirements of assisting children with ASD, and were in fact based on suggestions by teachers, and as such have been well received.

## A New Domain with Kaspar

**Fig. 10:**
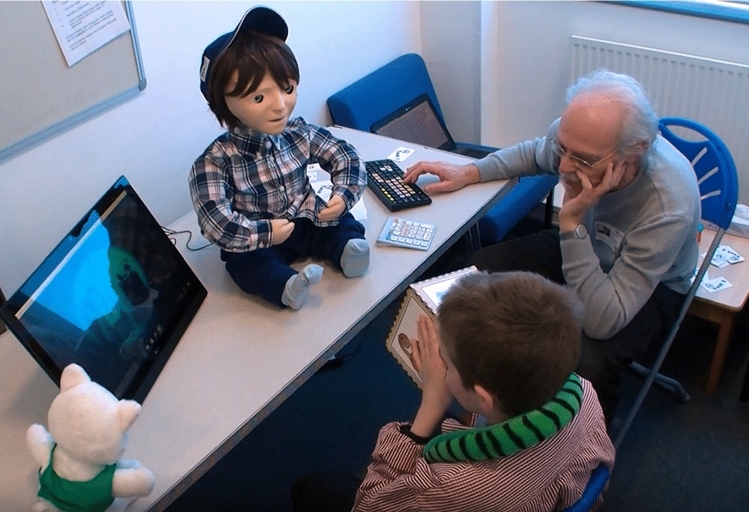
Child showing Kaspar an animal picture on the cube face

Recently our work with Kaspar has focused on developing the Visual Perspective Taking (VPT) skills of children with ASD [12, 42]. VPT is the ability to see the world from another person’s perspective. Flavell [43] defined two levels of perspective taking: VPT1, the ability to understand that other people have a different line of sight to ourselves and VPT2, the understanding that two people viewing the same item from different points in space may see different things. In attempting to devise an approach teaching children with ASD about VPT, we have been developing and testing games that involve the children moving toys into and out of the Kaspar robot’s Field Of View (FOV) (Fig. 10), or physically controlling the robot’s line of sight. The key to these games is giving the children the ability to see the world from the robot’s perspective thus assisting them in learning about VPT.

## Developing Autonomy to Improve Usability

Since the latest application area puts a particularly high cognitive load on the human operator it is important to develop methods of making the robot easier to control and allow the adult operator to focus on the child and not the robot. Very often in robot-assisted therapy for children with ASD the robot has to be partially or fully controlled remotely by an adult operator, whether this is a researcher, therapist, teacher or parent. Whilst using this method enables the adult operator to administer a highly personalised intervention focusing on high-level objectives such as developing the child’s social skills, it does require the operator to divide their attention between the child and the robot to ensure that the robot is responding appropriately to the child’s behaviour. Although this method is highly robust and does not require a complex control system for the robot, it is an unsustainable model for long-term interactions as the cognitive workload on the adult operator is very high [44]. In order to reduce the cognitive load on the adult operator and allow them to focus on the child it is essential to increase the robots level of autonomy. However, current sensing technologies and computing techniques are not sufficiently robust and accurate enough to provide consistent, stable, as well as personalised and flexible human–robot interactions with children with ASD. These limitations also present an ethical issue. If the robot is not reacting to the child in the correct way it may upset the child, causing them distress or even encourage the wrong type of behaviour. It is therefore currently logistically unviable to develop fully autonomous robots for this application area. There is also literature supporting the argument that a fully autonomous system is not suitable for this user group [45]. Taking these factors into consideration it would seem that currently the most logical course of action is to develop semi-autonomous systems that reduce the cognitive load on the human operator but still keep them in the control loop as this strikes the best balance between the current capabilities of technology and the application area. In respect of this the semi-autonomous system we devised for Kaspar has a degree of autonomy and as such only requires partial control by a human operator reducing the cognitive workload on the operator. In this approach the therapists, teachers or parents retains control over the robot’s high level behaviours to ensure the learning or therapeutic objectives are being met to deliver an effective treatment to the children with ASD.

**Fig. 11:**
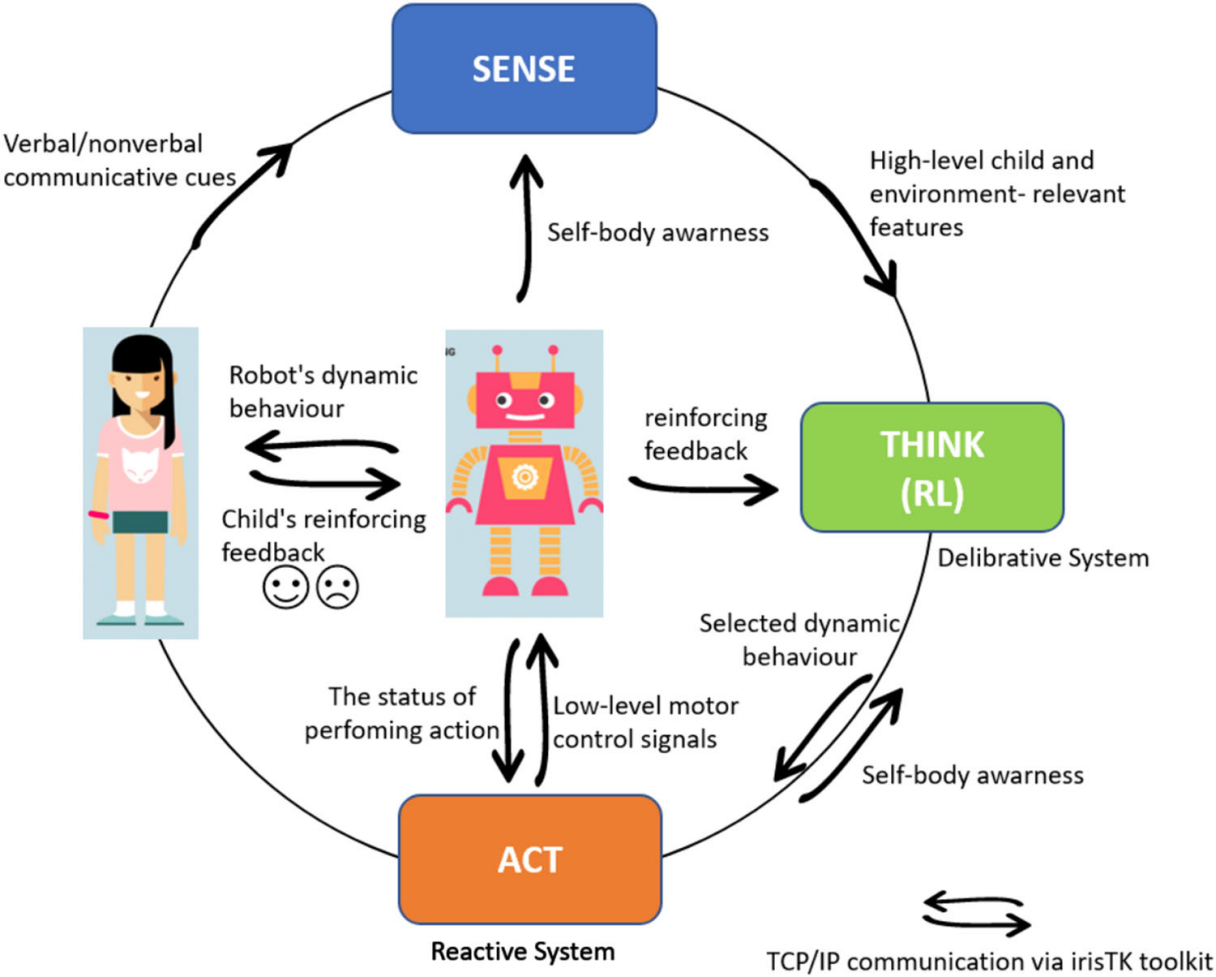
Kaspar’s deliberative-reactive control architecture (Sense-Think-Act) for semi-autonomous CRI

## A Deliberative-Reactive Control Architecture

To integrate some semi-autonomous features into the most recent generation of the Kaspar robot (K5.5) [46], Zaraki et al. have developed an interactive Sense-Think-Act architecture (Fig. 11) which has been employed to control particular aspects of Kaspar’s behaviour in a semi-autonomous manner [47]. The Sense-Think-Act architecture we have developed uses IrisTK [48] which is an event-based toolkit for real-time multiparty HRI. It consists of a message (event) passing system, a set of modules for multimodal input and output, and a dialog authoring language based on the notion of state-charts. Although the toolkit was originally designed for the Furhat robotics platform [49], we have adapted and integrated it for Kaspar’s architecture. In the context of the VPT games we developed, the system would effectively use the Sense-Think-Act architecture to sense the environment that was in Kaspar’s FOV. Then it would Think of what it should do next, depending on the object and position, then it would Act by suggesting a proposed behaviour to the operator for approval.

### Details of the Sense-Think-Act Architecture

**Fig. 12:**
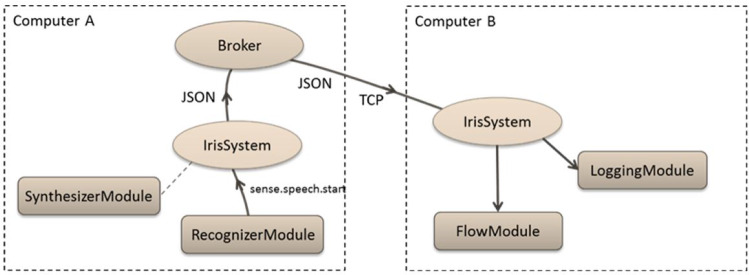
The IrisTK can be distributed over different processors and they can communicate the events via a central Broker. (this figure is taken from www.iristk.net)

**Fig. 13:**
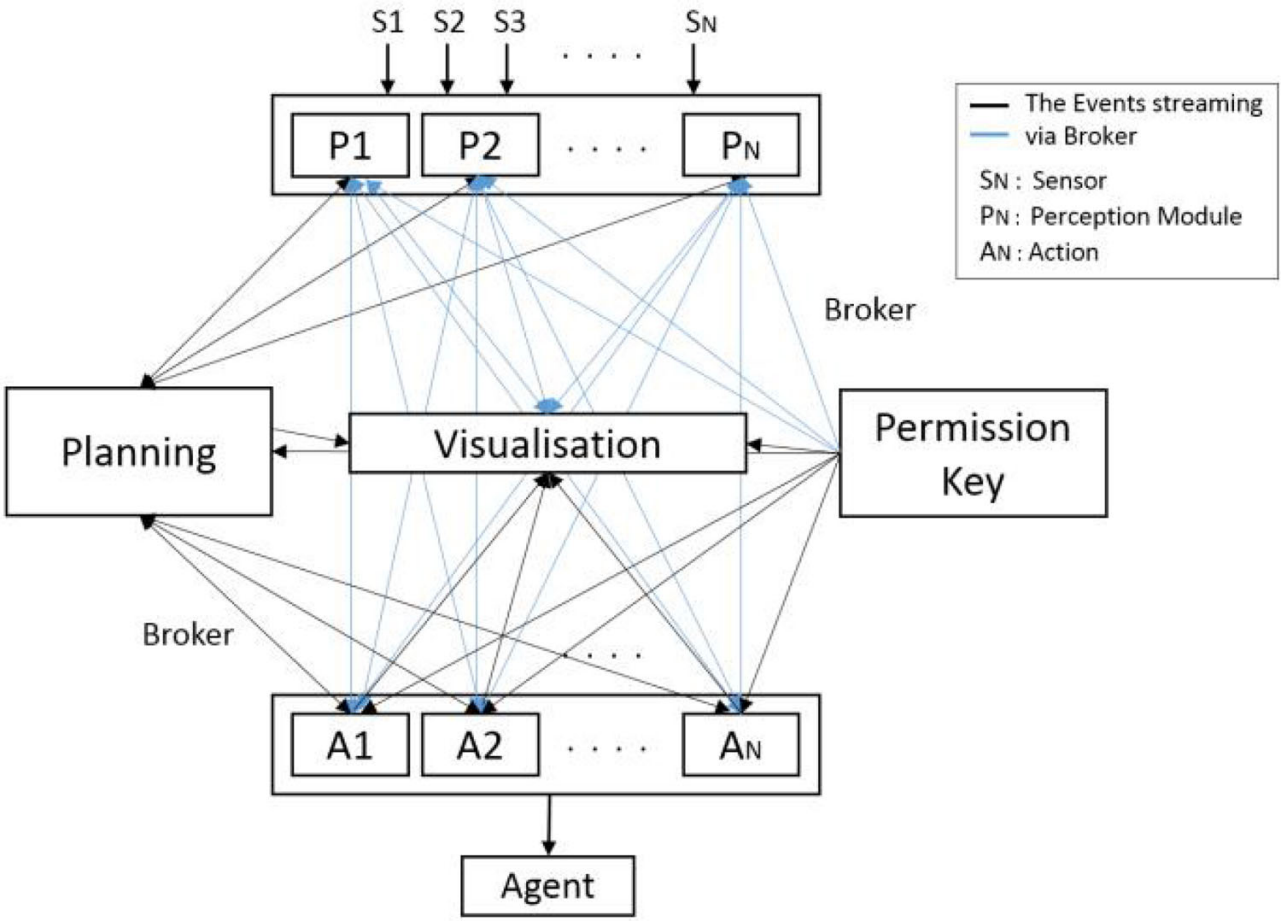
The Sense-Think-Act is a fully connected architecture. All the modules can send/receives the events via the IrisTK Broker (blue and black arrows), however there is the possibility to subscribe only to the specific events (black arrows)

The Sense-Think-Act architecture on Kaspar is a network-based platform independent architecture that has been implemented in a number of different programming languages (C sharp .Net and Java) and is capable of being distributed over a number of different processors in order to reduce the total computational cost. Since we have created the architecture on the basis of the IrisTK toolkit, the architecture has the capacity to operate on a number of different machines. In this instance, several IrisSystems are connected to a central broker (IrisBroker), which relays events to all connected systems. In each machine the events are serialised in standard JSON data packets and sent over TCP/IP. Using this method, it is also possible to connect modules that are implemented in other programming languages (Fig. 12). The architecture includes three standalone layers fully interconnected via a TCP/IP network (Fig. 11). Each layer has a number of modules that process either the sensory data captured by sensors/hardware or the high-level information that is distributed to the network as “events” in the standard JSON data packets. The layers and modules are fully interconnected and have the capacity to send and receive “Events” via the Broker over the network (Fig. 13). Thanks to the architecture’s modularity and network structure, the system is capable of running on multiple devices which facilitates handling of the overall processing cycles for real-time applications, if required. One of the primary benefits to this architecture is the potential for scalability allowing us to easily extend the architecture by adding new sensors/hardware devices and also new modules to the system. The architecture operates by collecting the sensory data and extracting high-level information then streams the corresponding “events” as JSON data packets to the networks (Sense Layer). The central layer receives the JSON packets and evaluates which reactive behaviour is the most appropriate for the current situation taking into account the interaction status and high-level information, and then streams an action “event” (behaviour name) to the network and asks the robot to display that behaviour (Thinks Layer). The Act layer receives the action event from the network and displays the behaviour on the permission of operator and returns the feedback/monitor “event” to the network to confirm that performing the action has been completed (see [47] for further details). Since the architecture communicates the events in JSON packets it is ideal for real-time HRI where the data communication is extremely quick with minimal lag time. Although the Sense-Think-Act architecture is fully interconnected meaning that the modules have the capacity to receive all the distributed events over the network. In order to reduce the computational costs, in each layer there is the possibility to subscribe only to those events that are necessary for that layer and dismiss all the other events (Fig. 13 black arrows).

### The Sense Layer

The sense layer includes a number of sensors which sense the environment and the associated perception modules that we have developed to interpret the sensory information in order to extract “events” occurring in the CRIs. We have chosen the sensors and developed the associated perception modules based on the requirements of the VPT games that we have been developing for the Kaspar robot. The core technical requirements to facilitate these games are object recognition and 3D orientation tracking.

#### Object Recognition

**Fig. 14:**
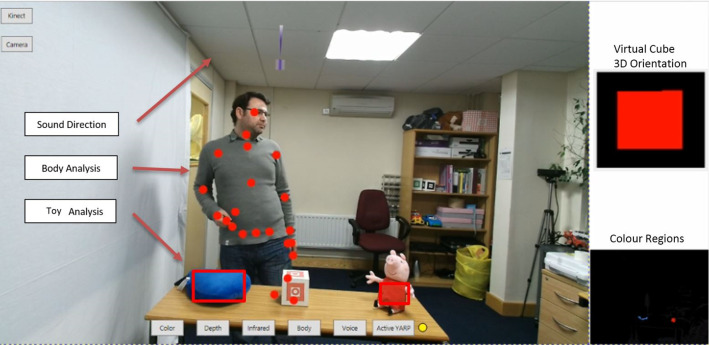
The visualisation of the objects 2D position and human body gesture detected by object analysis and human analysis modules

Since the core objective of the robot in the application area is to teach the children about VPT, it is essential that the robot has the capacity to robustly recognise an object if a useful level of autonomy is to be achieved. The perception module developed for Kaspar has been implemented using an image processing library (Aforge .Net) which receives the image from the embedded camera in Kaspar’s eyes and tracks and recognizes multiple toys based on their colours and the size of the colour regions (Fig. 14). In order to analyse the toys that the children brings into and out of Kaspar’s FOV, image processing techniques such as blob detection and colour filtering have been employed to detect and extract an object from the background and determine the pixel address in the 2D frame. For this reason, the object analysis module, firstly, acquires the image constructed by the RGB camera embedded in Kaspar’s eye, and processes the image in order to convert its specifications (dimensions and pixel ratios) into the one, required for the filtering step. The module then applies different filters to filter out the specific colours in order to identify the colour regions in the image. Finally, it returns as the output, the pixel address (x,y) for each object in the camera’s FOV.

#### 3D Orientation Tracking

**Fig. 15:**
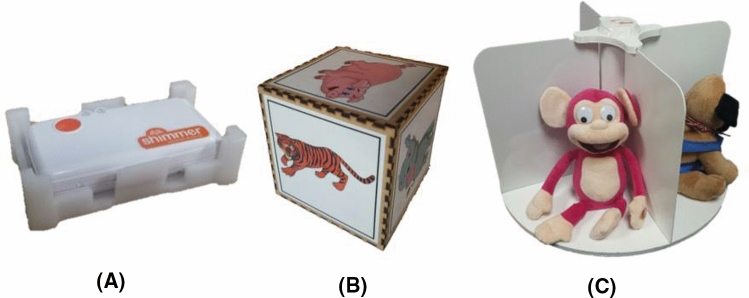
**a** The Inertial Measurement Unit (IMU) used to develop and sensorised cube and turn table, **b** the sensorised cube, **c** the sensorised turn table

Because some of the VPT games require an accurate representation of the objects orientation, Inertial Measurement Unit (IMU) sensors were used in some of the objects (Fig. 15) to estimate their position and orientation in relation to the robot. This has been achieved by analysing the accelerometer, gyroscope and magnetometer signals of the IMU. Analysing the data of the sensors of IMU through a regressive process, the module returns the 3D orientation vector of the IMU which is embedded in the cube and turn table which are used in the VPT games. Knowing the 3D orientation of the cube and turn table is very important since that enables the system to understand which side of the object is currently being observed by the child and which side is being presented to Kaspar. The main factor affecting the precision of the IMU data as well as the performance of the tracking algorithm is the calibration parameters. The three sensors of the IMU need to be calibrated prior to using it. The calibration process results in three matrixes called the calibration parameters which can be store in the IMU’s built-in memory. For the calibration of the IMU’s magnetometer the IMU needs to be rotated IMU around its three axes in different random directions until we see a nice aspherical shape in the calibration software GUI. For the calibration and the validation the standard Shimmer 9DOF calibration software provided by the manufacturer of the sensors was used.

**Fig. 16:**
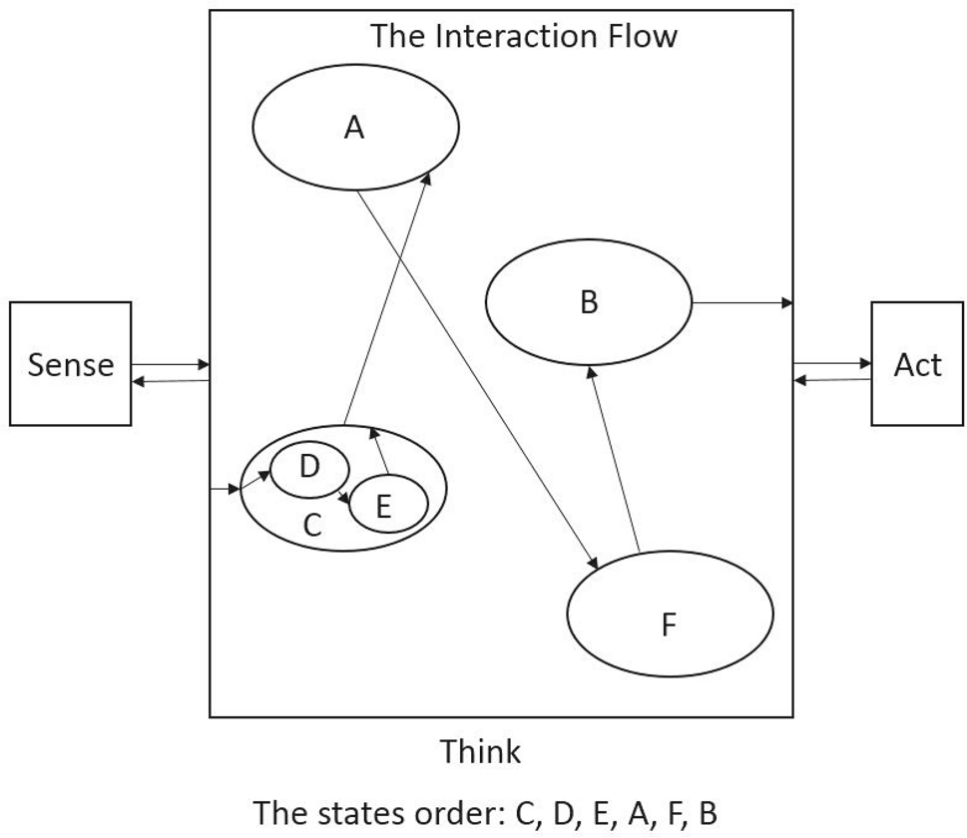
An example of the interaction flow of game 1 where the child must show the robot a toy of their choice

### The Think Layer

**Fig. 17:**
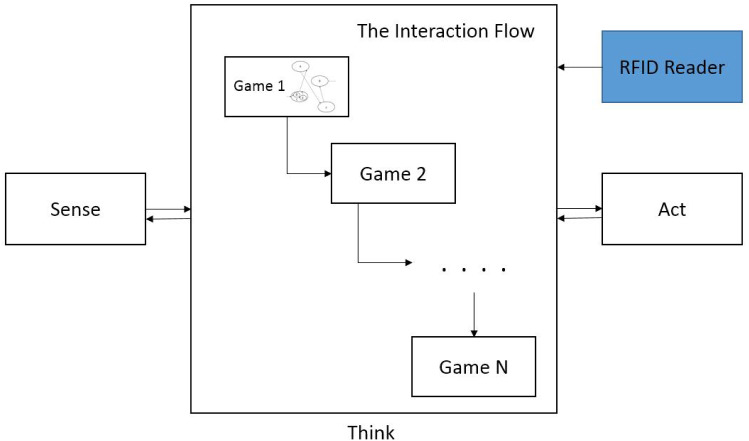
The implementation of the games in the deliberative module “Think” layer of the architecture

The Think layer is functioning as the brain of the architecture and as such receives all of the event streams from the Sense and Act layers via the network (IrisTK Broker) in real time and decides how to handle this information in order to make an appropriate decision for the robot’s action. In the decision-making process it considers the game modality (which game the robot is currently in), the status of the game (the progress of the child in the game), and the previous action shown by Kaspar. Figure 16 illustrates an example of the implementation of the games scenario in the Think layer. The interaction flow has an initial starting point for example state “C” in Fig. 16. The starting state could be an action such as the robot greeting the child or giving an introduction on the game to the child or whatever actions which encourage the child to engage and start the interaction with the robot. The Think layer activates the starting state once it has receives the relevant event/signal from the Sense layer for example a signal which shows child intention [50]. Using two main commands (“go to” and “return”), the interaction flow goes back and forth in the states to control the robot’s actions to procced with the game with the child in a way that will help the child to succeed in the game. For example the successive states could be for triggering robot’s action to provide positive/negative feedback to the child. The last states of the interaction flow could be to trigger the robot’s action to give a signal to show the game has been successfully completed or to ask the child to repeat the game if they would like to. In addition to the type of robot action in the interaction with the child, the dynamic of the robot’s action (such as gaze and attention) is controlled following the work presented in [51]. As shown in the Think layer, following the arrows, the states of the interaction flow that will be triggered are in the following order: “C (D>E), A, F, B”. The output of the Think layer is the name of the behaviour/action that system wants Kaspar to display and it is being sent directly to the Act layer. In fact, to keep the human operator in the robot’s control loop, the Think layer firstly shows the name of the behaviour to the human operator (on the screen), and Kaspar displays that behaviour only on the approval of the operator. Because a number of VPT games were implemented as different interaction flows, we have put all the flows in a single interaction Flow and allowed the user to choose the game which is preferable (Fig. 17). Therefore, prior to starting the game with the children the operator specifies the game number by scanning an RFID card to the system and afterwards the architectures will switch to that section of the Flow which is relevant to that game.

### The Act Layer

**Fig. 18:**
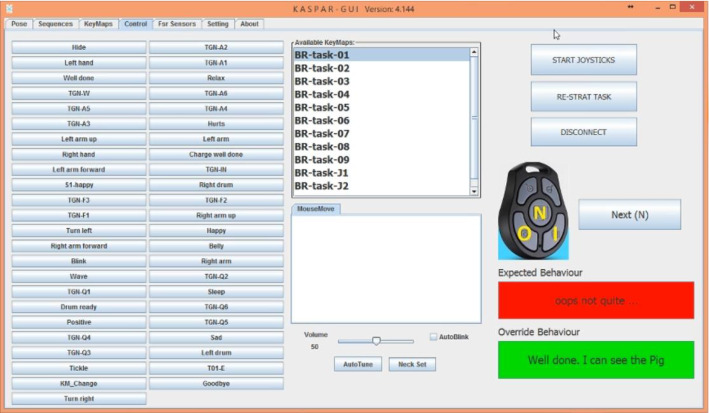
The reactive system (Act) layer of the architecture. The red box displays the behaviour that is suggested by the deliberative system and the green box displays the name of the correct behaviour that allows human operator to override the robot’s behaviour

The final layer of the architecture is a reactive (act) system which has been developed to provide Kaspar’s control signals in order to display different behaviours on the robot. to the IrisTK Broker and receives all the events however we have subscribed only to the Think layer which means that the reactive system only listens to the events that streamed from the Think layer. Similar to other layers, the reactive layer is also connected. The Kaspar reactive system (Fig. 16) has several pre-programmed behaviours stored as different external files that are typically used for generic play sessions and include various postures, hand waving, drumming on a tambourine that is placed on its legs and singing children’s rhymes. Each behaviour file includes the names of the sequences that are required to generate that behaviour. Each sequence file includes 22 motor position values to control Kaspar’s servos, and also the name of the voice files are to be played by Kaspar. With the previous Kaspar control architecture we were able to activate these behaviours by pressing a buttons either from a keypad or from the software interface. However in the semi-autonomous version, the Think layer will decide and activate a behaviour by sending an action event to the Act layer via the Broker. The Act layer has a sequence-player method that receives the name of the behaviour and plays the corresponding behaviour sequence. Figure 18 illustrates the Kaspar GUI for the reactive system which is connected to the architecture via the Broker. As shown there are two boxes (red, green) on the bottom-right corner of the GUI. The red box displays the behaviour that is estimated by the deliberative system according to the “perceptual information” provided by the Sense layer, and the green box displays the name of the correct behaviour that system estimates based on the “interaction status” and the “Interaction Flow”. These boxes will be shown on the GUI and the human operator has to make the final decision for the robot’s behaviour. The operator must give the final permission to the robot to display the behaviour presented in the red box or can override the robot’s behaviour and ask robot to display the behaviour presented in the green box.

**Fig. 19:**
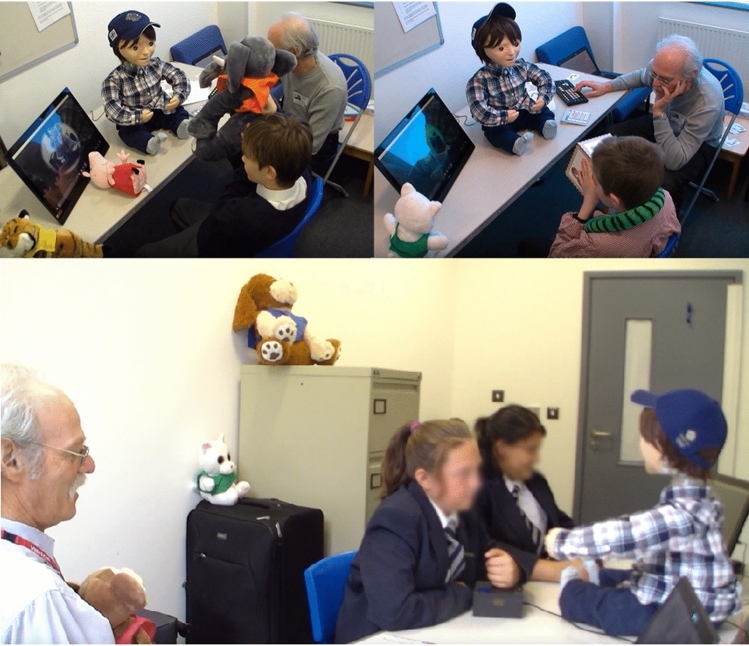
Trial with children with ASD in school

## Testing the Semi-Autonomous System

The semi-autonomous architecture implemented on Kaspar was tested at a school with children with ASD. Testing the system in this setting allowed us to evaluate the real-time performance of the architecture in controlling Kaspar in a real-world setting. It also allowed us to establish if the architecture is capable of controlling the robot’s behaviour in an autonomous and acceptable way in both dyadic and triadic interactions with children. We installed the three layers (“sense-think-act”) of the architecture on a single laptop (Toshiba Tecra, Intel Core i7, 2.60 GHz, 16GB RAM) for the compatibility test as well as to check the overall performance.

Four children with ASD that have different levels of ability took part in the study where they played 4 different games, one of which was a joint game for pairs of children. The games focused on VPT and included:

- Bringing the animal themed toys into the robot’s FOV
- Showing Kaspar animal pictures on different sides of a cube
- Physically manipulating Kaspar’s head to look at animal toys placed around the room
- Controlling the robot’s head orientation together via two joysticks (one controlling the horizontal movement, the other controlling the vertical head movement) as a pair to make Kaspar look at animal toys placed around the room.

During these sessions the data of 11 child–robot interactions were collected (Fig. 19). The trial showed that the architecture was capable of providing the robot with control signals in real-time without any latency throughout the duration of the sessions which provides evidence that the system is capable of supporting real-world applications. Although, the “think” and “act” layers functioned correctly in all of the interaction sessions, there were some real-world difficulties experience by the system (lighting conditions, etc.) which caused some problems in the object analysis module and subsequently had to overridden by the researcher. The three layers of architecture and their modules were successfully operated in real-time on the same laptop. The children were able to successfully play the games with the robot meeting the objectives of these games. This trial demonstrated that the architecture presented in this article was robust and did facilitate the outlined objectives but was not flawless due to the difficulties experienced by the perception system. Taking the lessons learned from this trial forward, our future efforts will focus on finding solutions to reduce or eliminate the problems experienced by the perception system in order to create a more robust system.

## The Wider Field of Robotics for ASD

Whilst our work with Kaspar has focused on developing both the hardware and software aspects of a robot with realistic humanlike features for children with ASD, many other projects have used other robotic platforms such as NAO [52], PROBO [53, 54] and Zeno [55, 56]. These platforms have been used in a number of projects that have also focused on working with children with ASD including the DREAM project [57], the DE-ENIGMA project [58], the SARACEN project [59] and many others. Whilst these projects are all working on valuable aspects of CRI for children with ASD, they are all very much focused on developing therapies with specific robotic hardware that is largely fixed. By contrast the approach taken with Kaspar, particularly in recent years is that the hardware can be designed and built around the needs of the users. For example, Kaspar can be physically manipulated by the children with no damage being caused to the servos of the robot which is currently unique to this platform. We can actively encourage children with ASD to touch the robot and manipulate its body parts. Furthermore the infrastructure of the new semi-autonomous system allows for additional sensory inputs to be included within the system, and these sensors do not need to be imbedded within the robot itself allowing for a much more flexible system preparing for future integration into smart environments where sensors could be placed all around the room.

## Conclusion

Since 2005 the Kaspar robot has continually been developed both in terms of hardware and software, during this time many lessons have been learnt about developing humanoid robots for children with ASD. The primary considerations that need to be observed when developing robots for this user group are as follows:

- *User focused*—Although technology can greatly assist in the development of robotic systems, it should not be the primary focus. The primary consideration should be the therapeutic and educational objectives rather than technology. Technology is merely a facilitator and should be used to fulfil the needs of the users.
- *Usability*—To ensure that technology has a genuinely useful impact on its target users it must be sufficiently usable, otherwise it will likely never be used and could even be seen as a burden by its users.
- *Reliability*—Instilling user confidence in a system is critical in getting users to want to use and embrace a system. Although this is particularly challenging in the field of assistive robotics for children with ASD, the Kaspar robot has been able to achieve good levels of reliability by considering how the users will use the system and what could and has gone wrong in the past. Developing any robotic system is an iterative process in order to make it reliable and thus embraced by users.
- *Safety*—Ensuring that any robotic system is safe is a top priority regardless of the user group. As such the Kaspar robot was developed to ensure that it was safe to use with children. This means ensuring that there were no pinch points, no chance of electrical shock, no sharp edges and numerous other considerations. The K5.5 robot was installed with extensive safety features to ensure it was suitable to be placed into a home or school environment.
- *Affordability*—In order for robotic systems to become accessible to users they must be produced at an accessible price. Ensuring that the Kaspar robot would potentially be affordable if it was to go into mass production has always been a key pillar of the platform and as such the latest K5.5 version of the robot has been produced with less than $\pounds 1600$ in components making it relatively cheap for such a complex mechatronic system.

As can be seen from the iterative development of the Kaspar robot over the last 12 years, technological advancements are enabling more useful and complex scenarios and systems to be developed. The advancements are not only facilitating new games that can assist children learn new skills, but are also making therapeutic robots such as Kaspar more robust. When Kaspar was first developed the ability to track users without attaching devices to them and with reasonable accuracy was not even a possibility. However, new sensing technologies such as the Kinect are not enough on their own. More work and research needs to be conducted in order to fully utilise the benefits of such technologies.
